# Wing Morphology, Foraging Strategies, and Flight Performance in Six Sympatric Species of Molossid Bats (Chiroptera: Molossidae) from Argentina

**DOI:** 10.1093/iob/obaf044

**Published:** 2025-11-14

**Authors:** M A Argoitia, P Teta, G H Cassini

**Affiliations:** División Mastozoología, Museo Argentino de Ciencias Naturales “Bernardino Rivadavia” (MACN-CONICET), Ángel Gallardo 470, C1405DJR Ciudad Autónoma de Buenos Aires, Argentina; Departamento de Ciencias Básicas, Universidad Nacional de Luján (UNLu), Ruta Nacional 5 y Constitución w/n, 6700 Luján, Argentina; Consejo Nacional de Investigaciones Científicas y Técnicas (CONICET), C1425FQB Ciudad Autónoma de Buenos Aires, Argentina; División Mastozoología, Museo Argentino de Ciencias Naturales “Bernardino Rivadavia” (MACN-CONICET), Ángel Gallardo 470, C1405DJR Ciudad Autónoma de Buenos Aires, Argentina; Consejo Nacional de Investigaciones Científicas y Técnicas (CONICET), C1425FQB Ciudad Autónoma de Buenos Aires, Argentina; División Mastozoología, Museo Argentino de Ciencias Naturales “Bernardino Rivadavia” (MACN-CONICET), Ángel Gallardo 470, C1405DJR Ciudad Autónoma de Buenos Aires, Argentina; Departamento de Ciencias Básicas, Universidad Nacional de Luján (UNLu), Ruta Nacional 5 y Constitución w/n, 6700 Luján, Argentina; Consejo Nacional de Investigaciones Científicas y Técnicas (CONICET), C1425FQB Ciudad Autónoma de Buenos Aires, Argentina

## Abstract

Differences in total wing area and shape have long been emphasized in relation to flight and foraging performance in bats. Molossid have a high species richness in the northern portion of Argentina, with many of them coexisting in sympatry and occupying very similar trophic niches. We characterize the wing shape and size of a molossid bat assemblage of six species from the humid Chaco region of northeastern Argentina. Considering that wing shape and size are good indicators of ecological and behavioral traits, we analyze the morphological variation using geometric morphometric tools. Our results provide information on changes in wing membrane morphology that are useful for explaining aspects of species sympatry. The variation in wing membranes is related to aspects of maneuverability, which consequently affects foraging strategies and prey (insects) capture. Also, this study serves as an example of the importance of applying geometric morphometric techniques in ecomorphological approaches.

## Resumen

Las diferencias en el área total del ala y su forma han sido foco de numerosos estudios en relación al vuelo y la búsqueda de alimento en los murciélagos. Los molósidos presentan una alta riqueza de especies en el norte de Argentina, muchas de las cuales coexisten en simpatría y ocupan nichos tróficos muy similares. Caracterizamos la forma y el tamaño de las alas de un ensamble de seis especies de murciélagos molósidos de la región húmeda del Chaco, en el noreste de Argentina. Dado que la forma y el tamaño de las alas son buenos indicadores de rasgos ecológicos y de comportamiento, analizamos la variación morfológica mediante herramientas de morfometría geométrica. Nuestros resultados aportan información sobre los cambios en la morfología de la membrana alar, útiles para explicar aspectos de la simpatría entre especies. La variación en las membranas alares se relaciona con aspectos de la maniobrabilidad, lo que a su vez afecta las estrategias de búsqueda y la captura de presas (insectos). Asimismo, este estudio resalta la importancia de aplicar técnicas morfometría geométrica en enfoques ecomorfológicos.

## Introduction

Most species of bats in the world are insectivorous belonging to the families Molossidae and Vespertilionidae (ca. 622 of the ∼1500 spp. sensu Mammal Diversity Database 2025). In particular, molossid bats have evolved flights that allow them to explore environments with different types of vegetation, from open to closed (e.g., jungles, grasslands, and forests). The first studies of bat flight in molossids characterized the flight performance of these animals mainly by wing area (Vaughan 1966; Norberg 1972). Small molossid species have more maneuverability, which allows them to capture prey in places where there are more obstacles, while most of the medium to large size species do so at higher altitudes where there are fewer obstacles (Norberg and Rayner 1987; Sánchez and Carrizo 2021).

Many molossid species have their southernmost distribution in the southern cone of South America, particularly in northern Argentina (Giménez and Giannini 2016). These species coexist in the same area, share their shelters, and occupies similar trophic niches. However, the adaptations that allow these species to coexist in the same assemblages remain to be elucidated (Argoitia et al. submitted). Particularly, in the northern portion of Argentina, there is a high species richness in sympatry, which can be attributed to the considerable heterogeneity of the environments present in these areas and their available resources (Argoitia 2023; Argoitia et al. submitted). Several studies have been assessed the differential habitat use among bat species within the same environment through ecomorphological approaches (e.g., Swartz et al. 2012; Marinello and Bernard 2014; Giménez and Giannini 2017).

Ecomorphological studies are a useful tool for assessing the relationship between organisms, their environment (e.g., correlations between behaviors and anatomy), and historical factors (e.g., ontogeny and phylogeny), to improve the understanding of biodiversity regulation (Rhodes 2002; Price and Schmitz 2016; Cassini et al. 2021). Ecomorphology is based on the premise that the coexistence of several species in a community implies the differential use of resources as the basis for niche differentiation (Hirst 1975; Jarman and Sinclair 1979; McNaughton and Georgiadis 1986; Castillo-Figueroa 2020). One significant feature of niche partitioning is size; when several taxa overlap in size, shape variation can be important (i.e., size independent; e.g., Jarman 1974; Bodmer 1989; Swartz et al. 2003; Cassini et al. 2012; Barbero et al. 2020; Vizcaíno et al. 2024). In this regard, the size and shape of structures in organisms are features that have been useful to test species differentiation (Struhsaker 1961; Findley et al. 1972; Aldridge and Rautenbach 1987), population variation (Saunders and Barclay 1992; Norberg 1994; Rhodes 1995), evolution of form and function (Cassini et al. 2021), and ecomorphology (e.g., Struhsaker 1961; Zhang et al. 2007; Maki and Fukui 2024; Ospina-Garcés et al. 2024).

Differences in total wing area and shape have long been emphasized in relation to foraging strategies and flight performance. The use of morphometric techniques, aimed at maximizing and detecting the morphological differentiation of bat wings, has been shown to be useful in understanding the dynamics of flight (Norberg 1981), segregation, and coexistence in space (Fenton 1972; Findley 1993). However, another important aspect on locomotion is the influence of the body size on animals; biomechanical studies have shown that as body shape changes with size, as do locomotor kinematics (Biewener 1983; Heglund and Taylor 1988; Biewener 2005). In these sense, animals of different sizes move their bodies differently because the physical demands of locomotion change with body size (Castillo-Figueroa and Pérez-Torres 2021; Almeida et al. 2025). Within the range of body sizes (from small to large), animal flight can differ in subtle but important ways. In contrast to insects and birds, it has largely been assumed that bats use similar aerodynamic force generation mechanisms in flight regardless of size (Norberg and Rayner 1987; Bullen and McKenzie 2002; Hedenström et al. 2007). Respect to shape changes, the proportion between wing membranes, curvature, and wing-tip are examples of variables that influence flight maneuverability, speed, and energetic costs (e.g., Norberg et al. 1993; De la Cueva Salcedo et al. 1995; Mancina et al. 2012).

The first studies in wing morphology proposed morphometric indices that take into account mechanics and aerodynamics to verify the flight performance of bat species that explore habitats differently and have different flight styles (Vaughan 1966; Norberg 1981; Freeman 1981a, 1981b). Area measurements and bone lengths are commonly used in bat morphometric (Farney and Fleharty 1969; Myers 1978; Williams and Findley 1979; Burnett 1983). For example, different flight actions and the length of the metacarpals are important for lift, whereas the length of the distal phalanges are important for maneuverability (Camargo and Oliveira 2012; Castillo-Figueroa and Pérez-Torres 2021). However, many times, these studies do not fully describe shape differences and neglect covariation of measurements or specific points in different regions of the wing (e.g., Birch 1995).

In recent years, photographs of bats with their wings fully extended have been analyzed using geometrics morphometric tools and different softwares (see Schmieder et al. 2015). These geometric morphometric methods have become popular because they allow the size and shape components of morphometric variation to be separated (Zelditch et al. 2012; Vizcaíno et al. 2024). The resulting variables are not redundant, and these approaches allow results to be visualized in terms of shape change, while maintaining the geometric properties of the objects throughout the analysis (Zelditch et al. 2012). In addition, these methods allow quantification of changes in the position of anatomical structures relative to each other, which are sometimes not captured by linear morphometric techniques (Schmieder et al. 2015). In contrast to this popularity in studies of other taxa, geometric morphometric has had scarcely use in studies of external morphology in bats (e.g., Birch 1997; Camargo and Oliveira 2012; Schmieder et al. 2015; Ospina-Garcés et al. 2024).

To assess the potential role of wing morphology in niche partitioning in bats, we studied a molossid bat assemblage consisting of six species inhabiting the humid Chaco region of northeastern Argentina. In addition, we explore how wing morphology relates to foraging strategy and flight capabilities. To evaluate this last point, we (1) characterized the shape and size variation of the wings via geometric morphometrics; (2) determined the variability of wing morphology within flight types; and (3) analyze the contribution of history (phylogeny) and ecology (flight performance) to observed morphological variation. We hypothesized that: (1) evolutionary history explains only part of the variation in bat wing morphology; (2) wing size varies across species of similar body mass (BM); (3) wing shape variation allowed different flight styles related to foraging behavior demands; and (4) niche partitioning among bat species depends on covariation in wing size and shape.

## Materials and methods

### Specimens and ecological data

A total of 202 adult individuals of both sexes (excluding females in advanced pregnant stage) of six species were studied: *Cynomops planirostris* (2), *Eumops patagonicus* (130), *Eumops perotis* (9), *Molossops temminckii* (3), *Molossus fluminensis* (12), and *Molossus molossus* (46) (see Appendix 1). Studied individuals are housed in the Colección Nacional de Mastozoología del Museo Argentino de Ciencias Naturales “Bernardino Rivadavia” (MACN-Ma). Molossidae bats are not easy to collect and some species are rare in the community, so sample size reflects success rate for capture using mist nets (method of collection of the animals analyzed) of bats in the assemblage’s study area and availability of museum specimens. This resulted in an imbalanced sample size, which could affect the interpretation of the results of our analyses, including intraspecific variation, particularly for certain species (see Cardini 2020 and references therein). Therefore, our analysis were performed on two data sets, which were then compared (see below): one including the total samples (six species) and another with only those species for which more than five specimens were available (i.e., a data set with four species).

The included species are known as fast-flying insectivorous bats, except for *M. temminckii*, which was recognized for its slow flight (Sánchez and Carrizo 2021). All specimens belonged to a peri-urban area of the city of Corrientes, Capital, Argentina (27°27′50.40′′S, 58°46′55.20′′W; 50 m. s. n. m.), from the Humid Chaco region (Burkart et al. 1999). This site is characterized by a heterogeneity of environments intermixed with human constructions, which include open spaces of modified natural grasslands, with presence of palm trees and more closed spaces of forest patches, with native and exotic trees.

### Wing shape and landmark data

According to Camargo and de Oliveira (2012), we have followed the same protocol for each specimen: they were thawed and then mounted ventrally on a styrofoam board with reference scales, using pins. The bat body was positioned at a 90° angle between the anteroposterior (snout-tail) and shoulder base-thumb axes. Subsequently, the wing position was standardized considering the maximum extension of stylopodium-zeugopodium and the wing membranes. A 14.2 Mp digital camera (Nikon D3100, 18–55 lens) was mounted on a tripod so that the lens axis was perpendicular to the wing surface to reduce parallax error. Photographic files were labeled with the acronyms and field numbers of each specimen for later digitizing landmark processing. This process was repeated three different times for each specimen of the total sample, to analyze a potential measurement error related to the variation of the mounting process. Prior to the digitization of the complete sample, training sessions were conducted to minimize variation due to setup. As a result, it was determined that for subsequent analyses, the consensus of three repetitions per specimen would be used.

**Fig. 1:**
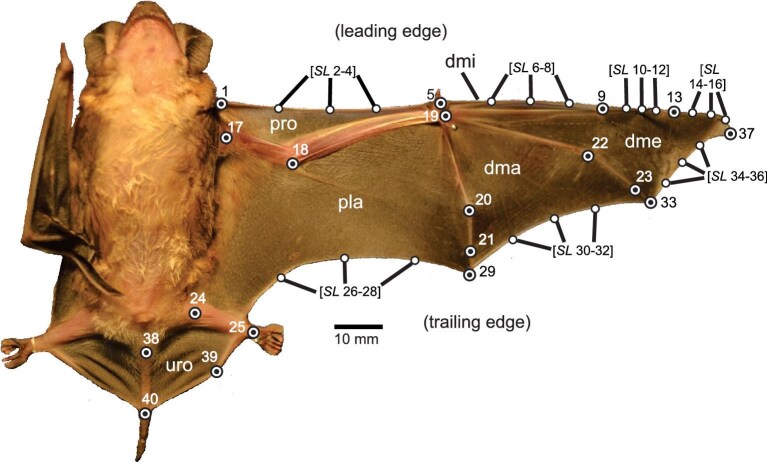
Example of wing mounting of *Molossops temminckii* used for the specimens studied. Landmarks and semilandmarks are shown with dots. References: ⦿ landmarks, ● semilandmarks. propatagium (pro), plagiopatagium (pla), dactylopatagium minus (dmi), dactylopatagium major (dma), dactylopatagium medius (dme), and uropatagium (uro). For landmarks definitions see Table 1.

The digitalization of landmarks was carried out using the thin plate spline (TPS) series software (i.e., TpsUtil v.1.83, TpsDig v.2.32) (Rohlf 2015). The landmarks are defined in Table 1 and shown in Fig. 1. They include 13 type I (anatomical tissue joints), 6 type II (maximum curvature points and calcar extreme), and 28 type III (semilandmarks on patagium leading and trailing edges) (see also, Camargo and Oliveira 2012; Schmieder et al. 2015). These anatomical landmarks aimed to provide a good representation of different wing regions (i.e., patagia, phallanxs, etc.) that can be easily identified in each specimen. In addition, the wing area, which consider the infolding of each individual wing membranes (i.e., plagiopatagium, propatagium, dactylopatagium minus, dactylopatagium major, dactylopatagium medius, and uropatagium) and the body mass data was taken from Argoitia (2023) to assess their relationship with wing shape.

**Table 1.** Anatomical definitions of the landmarks and semilandmarks considered for the molossid bats species studied

Point	Number	Definitions
L	1	End near shoulder of the propatagium membrane
*SL*	2–4	Anterior (leading) edge of the propatagium membrane
L	5	Tissue junction between the propatagium membrane and digit I
*SL*	6–8	Anterior (leading) edge of the dactylopatagium minus membrane
L	9	Articulation between metacarpus and proximal phalange of digit III
*SL*	10–12	Anterior (leading) edge of the dactylopatagium medius membrane
L	13	Articulation between proximal and intermediate phalanges of digit III
*SL*	14–16	Anterior edge of the dactylopatagium medius membrane
L	17	Base of the humerus
L	18	Articulation between humerus and radio/ulna
L	19	Center of the carpus
L	20	Articulation between the metacarpus and proximal phalange of digit V
L	21	Articulation between proximal and distal phalanges of digit V
L	22	Articulation between metacarpus and proximal phalange of digit IV
L	23	Articulation between proximal and distal phalanges of digit IV
L	24	Base of the femur
L	25	Union between the plagiopatagium membrane and foot
*SL*	26–28	Posterior edge of the plagiopatagium membrane
L	29	Tissue junction between distal phalange of digit V and the plagiopatagium membrane
*SL*	30–32	Posterior (trailing) edge of the dactylopatagium major membrane
L	33	Tissue junction on the trailing edge between distal phalange of digit IV and dactylopatagium major membrane
*SL*	34–36	Posterior (trailing) edge of the dactylopatagium medius membrane
L	37	Tissue junction between distal phalange of digit III and dactylopatagium medius membrane
L	38	Base of the tail
L	39	End of the calcar
L	40	End of the uropatagium membrane

### Geometric morphometrics

The landmark configurations were aligned using Generalized Procrustes Analysis (GPA), which superimpose the configurations and removes spatial variation that does not correspond to the shape applying rotation, translation, reflection, and scaling transformations (Rohlf 1990; Goodall 1991; Rohlf and Marcus 1993; Dryden and Mardia 1998, 1999). As a result, form is decomposed in its components, shape, and size [stored as centroid size (CS)]; see Goodall (1991). During GPA the geometric differences in scale are removed, but not the allometric components, so in the absence of allometry, CS does not correlate with shape (Bookstein 1986; Kendal 1986). Several authors have shown that CS is a geometric measure of size that follows the same mathematical behavior and is highly correlated with BM (e.g., Hood 2000; Ercoli and Prevosti 2011; Cassini et al. 2012).

### Phylogenetic signal

To assess the influence of phylogeny on both wing shape and size, a tree representing the phylogenetic relationships of the six of molossid species from the studied assemblage was constructed. In doing so, we used the dated tree resulting from the Bayesian analysis from Amador et al. (2018) and pruned the studied taxa using *drop.tip* function from ape v.5.8–1 R package (Paradis and Schliep 2019). We use the *K*-mult statistic, a multivariate extension of Blomberg’s Kappa‐statistic (Blomberg et al. 2003; Adams 2014) to evaluate the phylogenetic dependence of both wing shape and size morphogeometric data, and a given continuous trait (i.e., BM and wing area). Significance was assessed using 10,000 permutations. A *K*-mult value of 0 implies no phylogenetic bias, whereas a value of 1 means adherence to the Brownian motion or neutral model of character evolution. In addition, the phylogenetically aligned component analysis (PACA; see Adams and Collyer 2019), based on the ordinary least square (OLS) residuals from phylogenetic signal on species mean aligned Procrustes shape variables was stored to compare with principal component analysis (PCA) analysis (see below).

### Size and allometry

We used a phylogenetic generalized least squares (PGLS) regression analyses (Revell et al. 2012) to perform phylogenetic comparative analyses on the relationship between mean species shape and centroid size. The function *procD.pgls* from geomorph v.4.0 R package (Adams et al. 2024) was applied using the model: shape ∼ log 10 (CS) and shape ∼ log 10 (CS) + fly group (guilds). Significance was assessed using 10,000 permutations.

In order to evaluate the component of allometric shape variation including the intraspecific component, multivariate regressions of the landmark coordinates of the aligned specimens were performed against different size variables. The log 10-transformed CS (i.e., a proxy of overall wing size) and BM in grams was used to compare different components of shape variation.

The relationship between the area of the supporting surfaces of one wing of the two wings and the CS was evaluated by a Standardized Major Axis (SMA) regression. The wing area grows at quadratic rates, while CS does so linearly, so the two variables were log 10-transformed (logW and logCS), converting the relationship of these variables from a power formula to a straight line. The SMA approach is more appropriate for dealing with allometric approaches than OLS regressions (for an extensive overview on the subject see Warton et al. 2006). As a first step, the significance of the allometric coefficient was evaluated using a two-tailed *t*-test at a *P* = 0.05 level. Deviations from isometry were compared with the expected allometric coefficient under geometric similarity (Alexander 1985). An *F*-test was conducted to evaluate significant deviations from isometry (Warton and Weber 2002) with the null coefficient fixed at 2.0. A coefficient value <2.0 was considered negative allometry, while a coefficient value >2.0 was considered positive allometry.

### Multivariate morphometric variation

PCA was employed to visualize changes in shape concerning the distribution of taxa in morphospace and to identify the main components of variation (Mitteroecker and Bookstein 2011; Mitteroecker et al. 2013). To determine the number of interpretable PCs, the method proposed by Bookstein (2014) was applied. If the ratio of a PC to its successor exceeded a threshold based on a sample size dependent log-likelihood ratio, then the PC was considered to be interpretable. In both morphometric analyses (PCA and allometric regressions), the results produce vectors in shape space. An angular comparison was used to compare vector directions and examine the similarity between the allometry regression and the PCA vectors. In order to test the degree of covariation between this PCA and PACA, we performed a Procrustes correlation test between the centroid of species scores of interpretable PCs obtained previously, using the *procrustes* and *protest* functions of the vegan v.2.6–6.1 R package (Oksanen et al. 2022).

In each analyses, the phylomorphospace were superimposed using the *phylomorphospace* function of phytools v.0.7–80 R package (Revell 2012) in order to visualize the relative amounts of divergence vs. convergence among taxa (see Stayton 2015). All geometric morphometric analysis, visualizations, and graphs were conducted using the Morpho v.2.9 R package (Schlager 2017) under R v.4.4.2 environment (R Core Team 2023).

### Congruence analyses between data sets

We performed a Procrustes correlation test on a symmetric rotation to assess the degree of congruence between the analyses, including the intraspecific variation on wing morphology. Procrustes symmetric rotation uses uniform scaling (expansion or contraction) and rotation to minimize the squared differences between two ordinations (see Cassini 2013 and references therein). Using the *procrustes* and *protest* functions of the vegan R package, we compared the correlation between the scores of shared species in the morphospaces obtained from the shape analyses performed on both datasets (i.e., four and six species). Additionally, we computed the Procrustes residuals for each species. In the regression and PCA regression morphometric analyses, the results produce vectors in shape space. We compared the corresponding shapes of the extreme values (i.e., negative and positive most) produced by these shape change vectors using deformation of TPS gridlines plus wireframe.

## Results

### Phylogenetic signal

The multivariate generalization of Kappa statistic only yielded a significant phylogenetic signal for wing shape (*K*-mult ∼ 1.096, *P*-value = 0.01495; Table 2). The observed value above 1 indicates a high bias and adherence to the Ornstein–Uhlenbeck (OU) or one-dimensional random walk with a central tendency (i.e., a stabilizing force) of character evolution.

**Table 2.** Phylogenetic signal tests for shape, size, BM, and wing area data sets

Parameter	Wing shape	Wing log10(CS)	log10(BM)	log10(wing area)
*K*-mult	1.09585	1.00642	0.81638	1.00415
*P-*value	0.01495*	0.18315	0.49455	0.18665

Abbreviations: *K*-mult, multivariate generalization of the Blomberg’s Kappa-statistic; CS, centroid size; BM, body mass in grams; and area, total wing area in mm^2^. Asterisk, significant after 10,000 rounds of permutation test.

### Allometry

The PGLS analyses yielded a marginal but significant (*P*-value 0.0497) relationship between species mean log10-transformed CS and shape coordinates. However, when flight modes are added to the model, the effect of CS, but not flight guild, was significant (*P*-values 0.02475 and 0.07895, respectively; see Supplementary Material 1, Table 1 and Fig. 1).

**Fig. 2:**
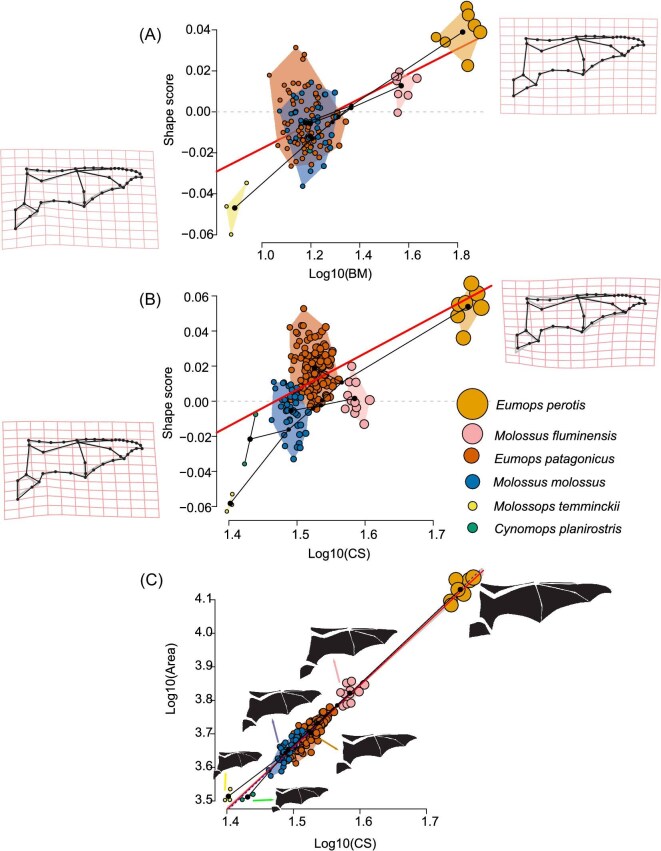
Multivariate regression analysis for the six species of molossid bats. (A) OLS regression of Procrustes coordinates (shape scores) versus log10-transformed BM; (B) OLS regression of Procrustes coordinates (shape scores) vs. log10-transformed CS; (C) SMA regression of log10-transformed total wing area (mm^2^) vs. log10-transformed CS. Deformation of TPS gridlines plus landmarks and wireframe (gray, the overall mean shape; i.e., consensus) of negative and positive most shape score values are exaggerated three times.

Both regressions of shape against log10-transformed CS (log10CS) and body mass (logBM) were significant, explaining 7.2 and 6.86% of the variation in shape (allometric scaling) with total sum of squares = 0.3259 and 0.223, respectively. In both morphospaces, the species were distributed along a gradient with the smallest on the negative values to the largest on the positive values of the *y*-axis (shape score) (Fig. 2A and B). They also share the allometry pattern in which the angle between both change vectors in the 39.08°, *P*-value <0.0001.

In the logCS regression analysis of shape, the species showed a good fit to the regression line. Shape changes showed an allometric trend, mainly from shorter wings to greater wing in relation to changes in the elongation of the propatagium, plagiopatagium, and uropatagium (Fig. 2A). The morphospace of the regression showed a gradient from left to right (*x*-axis) with increasing logCS of the wing. In this morphospace, *E. perotis* lies at the positive extreme of the line, well separated from the other species, with a morphology characterized by narrow membranes with less surface area than the small species *C. planirostris* and *M. temminckii* with wide membranes and large surface area. Intermediate size values were found for *Molossus fluminensis, Molossus molossus*, and *E. patagonicus*. In the case of *E. patagonicus* and *E. perotis*, they were also found above the regression line, which indicates a great variation in the shape of these species in relation to their size.

On the other hand, in the logW regression of shape, the morphological membrane changes were similar to those observed in logCS regression (Fig. 2B). In the morphospace, the polygons delimiting the intermediate size species *Molossus molossus* and *E. patagonicus* showed a marked overlap with a *C. planirostris*, sharing the same space over the line. This indicates that the three species are similar in relation to BM but have high disparity in terms of shape. In particular, *E. patagonicus* showed the highest variation and was found above the regression line (as in the case to log10CS) indicating the high correspondence of shape changes in relation to their BM values. It is noteworthy that *Molossus fluminensis* has similar scores for the positive *y*-axis (i.e., shape score) as *Molossus molossus, E. patagonicus*, and *C. planirostris* but differentiated in logW. With respect to *M. temminckii*, remained at the negative extreme characterized by its small size and BM.

The results of our morphogeometrics analyses do not change even if the analysis is repeated using the subset of four species. In fact, the high Procrustes correlation and the very small Procrustes mean residuals by species indicate a very high congruence (see Supplementary Material 2 for further descriptions).

The analysis of SMA of the relationship between log10-transformed wing area and log10CS showed that all coefficients were significant (Fig. 2C). A very high coefficient of determination was obtained (*R*² = 0.978; *F* = 3869.126; *P*-value <0.0001) with a slope of 1.87 ± 0.06 (95% confidence interval). The isometry test indicated a significant negative allometric trend (*F*_iso_ = 11923.95; *P*-value <0.0001), meaning that the larger species *E. patagonicus* showed proportionally lower total wing area values than expected for its CS. This highlights that while CS may be a good predictor of wing area, there are size-related differences observed.

### Multivariate shape variation

**Fig. 3:**
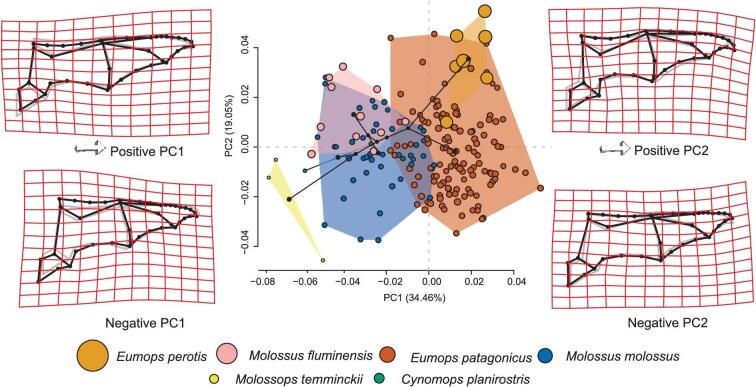
PCA for the six species of molossid bats. Morphospace of the first two principal components (PC1 and PC2). References: the size of the points are proportional to the CS and TPS gridlines plus landmarks and wireframe (gray, consensus; i.e., zero values) of negative and positive most first pair of PCs.

The PCA defined by the variation components of the wing membranes indicated that the first four components explained ca. 69% of total variance and they showed to be meaningful after Bookstein´s (2014) method. The components PC1 and 2, explained ca. 54% of total variance (Fig. 3) and showed information related to changes in the shape of the wings. These changes mainly occurred in relation to differences in the proportions of metacarpals and proximal phalange of fifth digit, wing tip and the extent of the membranes of the uropatagium, propatagium, and minor dactylopatagium. The first component (PC1) accounted for 34.46% of the variance. Toward the negative values, the proximal phalange of fifth digit is ca. 0.4, the length of metacarpal, dactylopatagium minus, major, and medius are more rounded at the wing tip with a size similar in proportion to that of the arm patagium, and there is a greater extension of the propatagium, and a broader uropatagium. Toward the positive values of PC1, the proximal phalange of fifth digit is ca. 0.6, the length of metacarpal, and the elongated wings were primarily related to a great width of the dactylopatagium major, a larger extension of the plagiopatagium in relation to the propatagium, and a less developed uropatagium (Fig. 3). The second component (PC2) explained 19.05% of the variance. Toward the negative values, wing shape are characterized by pointed wingtips, a straight wing leading edge of the propatagium, a large chord length, great extension of the plagiopatagium, and a narrow chest. Toward the positive values of PC2, there was a curved wing toward the trailing edge, a plagiopatagium with a more pronounced curvature of the posterior edge, small chord length, and width chest (Fig. 3).

In the morphospace depicted by PC1 and 2 (Fig. 3), the smallest species (i.e., low elongation and wing load), *M. temminckii*, separated toward both negative extremes, while the bigger one (i.e., great elongation and wing load), *E. perotis*, occupied the opposite end. The species *Molossus molossus* and *E. patagonicus* expanded widely and occupied an intermediate position in morphospace, but showed differences between them regarding their occupation of PC1, whereas the remaining species showed differences concerning PC2. The second quadrant was occupied by *Molossus fluminensis* and *C. planirostris*, which were very close, but with a tendency for the latter to occupy the double negative quadrant.

The PC3 and PC4 explained a small percentage of the variance, 16% (PC3: 10.37% and PC4: 5.5%). Shape changes were related to the arm patagium that ranged from width to narrow from the positive to negative extremes of PC4 and to dactylopatagium minus, major, and medius from width to narrow from the positive to negative extremes of PC3 (for PC3 and PC4 graphs and their respective shape and size changes, see Supplementary Material 3). The fourth quadrant segregated *M. temminckii*, while all other species were overlapped and close together, occupying a large extent of the morphospace. These results keep almost similar if the analysis is repeated using the subset of four species. Although some rotations between the morphospaces are required, they showed a high Procrustes correlation, a very small Procrustes residuals and a negligible difference between shape vectors (see Supplementary Material 3).

The Procrustes superimposition of species centroids from the multidimensional morphospace of these four PCs eigenvectors and the corresponding components of PACA based on OLS residuals, rendered a 0.9835 correlation for the symmetric Procrustes rotation (*P*-value = 0.00139; after 10,000 rounds of permutation test). The taxa with the largest residuals, indicating the parts of the morphospace that experienced more changes between these two analyses were *C. planirostris* (ca. 0.01126) and *M. temminckii* (ca. 0.00893), while *E. patagonicus* showed the lowest residual (ca. 0.00368).

## Discussion

Our results provide information on the changes in the size and shape of wings of molossid bats. Besides that shape coordinates showed some phylogenetic signal, they are still useful for explaining aspects of sympatry among morphologically similar species. The studied assemblage serves as an example of the importance of applying geometric morphometric techniques in ecomorphological studies.

Size was the more significant feature for niche partitioning in the studied molossid bat assemblage. The three regression analyses clearly separated small species with low wing elongation and BM (i.e., *M. temminckii*) from the larger ones, that have larger wings and BMs (e.g., *E. perotis*). In addition, our results also depict the variation in relation to shape of curvature of the membranes, principally for the dactylopatagium minus, uropatagium, and propatagium. These membranes are related to the control of maneuverability, the edge, and the angle of attack of the wing profile during flight, which consequently affects foraging strategies and insect prey capture (e.g., Dudley 2002; Gardiner et al. 2011). All these findings correspond positively with the proposal hypothesis that size and shape wing covariations could explain a separation of trophic niche.

The CS of the analyzed landmark configuration proved to be a good predictor of wing area. Indeed, both variables did not have significant phylogenetic biases. These results validate our first hypothesis on the partial influence of evolutionary history in wing morphology variation (i.e., only shape but not size). The observed negative allometry suggest that species with a larger CS have a smaller area than expected under an isometric relationship. By comparing the regression lines, it was evident that shape changes in species of similar BM (i.e., *C. planirostris, E. patagonicus*, and *M. molossus*) compensate for wing load values. This allows validate our second hypothesis, which depict that wing size vary across species of similar BM, primarily on their total area. Also, it is clear that the differences between species of similar BMs are due to differences in wing shape related to changes in the curvature of the wing membranes (given support to our third hypothesis). The body size and wing surface area (i.e., wing loading see Norberg and Rayner 1987) are closely related to the maneuverability fitness due to the changes involved to different wing loading in species of similar size. Thus, species of similar size may have variations in their maneuverability capabilities related to differences in area due to shape changes. Another aspect that was evident was the different sets of wing elements (i.e., metacarpals, proximal, and distal phalanges). This was reported in other studies, such as those of Stevens and Guest (2022), that related bones elements of wings to different aspects of the performance of flight. The variation in fifth phalanges observed by us corresponds to their proportion relative to the mean chord and their relation to long wings, flight velocity, and energy costs (see similar results in Findley et al. 1972; Dietz et al. 2006; Castillo-Figueroa 2020). In general, phalanges are the primary propulsive portion of the chiropteran wing, whereby longer wingtips are related to greater speed (Findley et al. 1972; Altringham 1996). On the other hand, metacarpals are important for generation of lift (Findley et al. 1972), while relative length of metacarpals defines the degree to which curvature of the wing is located toward the trailing edge of the wing, which influences maneuverability (Stockwell 2001).

Our results highlights the different dimensions of changes that can occur in wing morphology between similar species that co-exists in the same area. In relation to the body size, these aspects have been used in many studies, explicitly in relation to the effects on a wide range of behaviors, such as activity levels, dominance, home range size, competitive ability, foraging ecology, sexual selection, predation risk, reproductive tactics, and parental care (e.g., Hedenstrom and Rosen 2001; Brown and West 2000; Roff 2002; Dial et al. 2008).

Bats use different flight styles and foraging techniques related to their feeding behavior. Particularly, flying insectivorous bats depend on their agility (ability to initiate a turn quickly) and maneuverability (ability to turn tightly) to capture prey (Norberg 1986; Norberg and Raymer 1987). Both aspects of flight performance decrease with increasing body size (Aldridge 1987; Norberg and Rayner 1987). In terms of maneuverability, wing area does not increase proportionally as wing loading (i.e., BM/total wing area) increases, forcing large bats to fly faster to maintain lift, thereby reducing maneuverability (Norberg 1986; Norberg and Rayner 1987). Accordingly, bats have lower wing loadings than similarly sized aerial insectivorous birds and are therefore more maneuverable (Norberg 1986). Thus, aerodynamic constraints do not appear to limit the body size of aerial insectivores relative to their avian counterparts.

The functional structure of the molossid assemblage shown in the PCA analyses was mainly influenced by the size of the species and by differences in the extent of the wing membranes. The morphofunctional segregation observed in the PCA occurred primarily along an interspecific gradient that responded to size and shape mixed effects (see Fig. 3). In morphospace, a diagonal cline with the smaller specie *M. temminckii*, located at the negative extremes of PC1 and PC2, while the largest species, *E. perotis*, was positioned at the opposite extreme. This morphospace appears to be structured phylogenetically, with the *Molossus* and *Eumops* genera occupying distinct regions and showing little overlap. This arrangement, along with the few crossings of the phylogenetic tree branches projected into morphospace, is consistent with the phylogenetic signal found in wing shape. Within each genera, the species of bigger sizes (i.e., *Molossus fluminensis* and *E. perotis*) tend to lie at higher positive values of PC2. In addition, *C. planirostris* clusters with *Molossus* spp. rather than their sister species *M. temminckii*, showing a convergence in the size and shape mixed effects of wing morphology.

The effect of size on the structuring bat assemblages has been reported by numerous studies (e.g., Marinello and Bernard 2014; Giménez and Giannini 2017; Magalhães de Oliveira et al. 2020; García-Herrera et al. 2023). Such variation may in turn, therefore, lead to niche differentiation and specialization due to individuals having different food preferences, with a consequent expansion of the overall dietary niche (Svanbäck and Bolnick 2007). Also, Giménez and Giannini (2016) suggested that the effects of size, along with history and functional morphology, drove the evolution of neotropical molossid bats and facilitated the coexistence of these species. Some studies have shown that in sympatric Old World rhinolophid bat species size and echolocation calls mainly drives niche partitioning relative to diet (see Dai et al. 2023 and references there in). Alternatively, they showed to be more divergent in the use of foraging habitats than diet, and so, seems to be the main mechanisms for resource partitioning putatively due to differences in wing morphology (Salsamendi et al. 2012). Following Schmieder et al. (2015), size and shape covariation, instead of wing morphology, seems to better explain the flying foraging performance in different environments. Accordingly, our results validate our fourth hypothesis about the influence of wing size and shape morphology in the niche partitioning of species, agreeing with Schmieder et al. (2015). Aside from a clear effect of size, all analyses performed here preclude separating the effects of both size and shape and lean in favor of a covariation in wing size and shape as the source in niche partitioning.

The correspondence of size and shape effects with aspects of sexual dimorphism for each species could explain the intraspecific variation. Future studies with larger samples and similar male to female proportions could focus on identifying significant variations between sexes. In addition, the guild categories used here oversimplify the flight styles related to foraging behavior demands and diet. However, in order to better analyze the correspondences between flight styles and foraging behavior with wing morphology, further exhaustive analyses at macroevolutive scale need to be achieved (see for example the approximation in defining diet categories followed by Gagnon and Chew 2000 in bovids; Olsen 2015 in anseriformes birds; Barbero et al. 2021 in sigmodontine rodents; Cassini and Toledo 2021 in cervids; Sánchez and Carrizo 2021 in bats).

We demonstrate that geometric morphometric analyses are a very powerful tool for studying the assemblage organization and foraging performance of bats, allowing the understanding of ecological attributes that are difficult to observe directly under natural conditions. However, it is necessary to stress the importance of carrying out ecological studies on aspects such as feeding behavior, activity patterns, roost use, and bioacoustics, (among others), especially for the bat species of the Southern Cone of South America, whose behavior is still little known. Finally, we emphasize the relevance of applying geometric morphometric techniques to redefine or specify in a more thorough manner the designation of types of flight to explain relationships of bats sympatry. At the same time, it is important to highlight the complexity involved in understanding the dimensions in which the structuring of a bat assemblage can be understood and explained in relation to its functionality and trophic aspects. This study is one of the few to utilize this type of technique for bats within southern South America, as until now these tools had seen limited use in aspects of the external morphology of this group in relation to other mammals.

## Supplementary Material

obaf044_Supplemental_Files

## Data Availability

The data underlying this article will be shared on reasonable request to the corresponding author.

## Appendix I

Specimens of molossids bats species studied. Total number (*n*=) by species, field acronym (AMA), or collection acronym of Colección Nacional de Mastozoología of Museo Argentino de Ciencias Naturales “Bernardino Rivadavia” (MACN-Ma), followed by specimen number and sex. Asterisk, indicated individuals with BM data available, ♀ for females and ♂ for males.

*Cynomops planirrostris* (*n* = 2) ♀: AMA 226*. ♂: MACN-Ma 31159*.

*Eumops patagonicus* (*n* = 130) ♀: AMA 14, AMA 17, AMA 18, AMA 23, AMA 34, AMA 37, AMA 42*, AMA 44, AMA 53, AMA 57*, AMA 59*, AMA 62*, AMA 70*, AMA 71*, AMA 73*, AMA 76*, AMA 78*, AMA 79*, AMA 80*, AMA 83*, AMA 84*, AMA 87*, AMA 88, AMA 93*, AMA 94*, AMA 95*, AMA 100*, AMA 101*, AMA 102*, AMA 105*, AMA 113, AMA 118, AMA 122*, AMA 125*, AMA 127*, AMA 134*, AMA 135*, AMA 136*, AMA 137*, AMA 139*, AMA 147*, AMA 149*, AMA 157*, AMA 162*, AMA 163*, AMA 164, AMA 166*, AMA 169*, AMA 172*, AMA 175*, AMA 176*, AMA 179*, AMA 180, AMA 183, AMA 186, AMA 187, AMA 188, AMA 192, AMA 193, AMA 195*, AMA 196, AMA 198*, AMA 199, AMA 200, AMA 202, AMA 204, AMA 206, AMA 209, AMA 210, AMA 212, AMA 215*, AMA 216*, AMA 231. ♂: AMA 8, AMA 9, AMA 22, AMA 24, AMA 27*, AMA 28*, AMA 36, AMA 43, AMA 45, AMA 56*, AMA 58*, AMA 60, AMA 64*, AMA 68*, AMA 85*, AMA 86*, AMA 91*, AMA 92*, AMA 97*, AMA 98*, AMA 99*, AMA 103, AMA 115, AMA 119, AMA 123*, AMA 124*, AMA 126*, AMA 132*, AMA 133*, AMA 140*, AMA 141*, AMA 144, AMA 146*, AMA 152*, AMA 156*, AMA 158*, AMA 159*, MACN-Ma 31192*, AMA 161, AMA 165*, AMA 167*, AMA 168*, AMA 171*, AMA 173*, AMA 174*, AMA 177*, AMA 178*, AMA 181, AMA 182, AMA 184, AMA 189, AMA 190, AMA 197*, AMA 200, AMA 201, AMA 203, AMA 205, AMA 207, AMA 208, AMA 211, AMA 213, MACN 31197*, AMA 218*.

*Eumops perotis* (*n* = 9) ♂: MACN-Ma 31135*, MACN-Ma 31137*, MACN-Ma 31139*, MACN-Ma 31140*, MACN-Ma 31141*. ♀: MACN-Ma 31136*, MACN-Ma 31138*, MACN-Ma 31142*, MACN-Ma 31143*.

*Molossops temminckii* (*n* = 3) ♂: MACN-Ma 31156*, MACN-Ma 31158*, MACN-Ma 31160*.

*Molossus molossus* (*n* = 46) ♀: AMA 4, MACN-Ma 31203, AMA 6, AMA 10, MACN-Ma 31190, AMA 13, AMA 15, MACN-Ma 31187*, AMA 26*, MACN-Ma 31206*, AMA 53*, AMA 54*. ♂: AMA55*, AMA 61*, AMA 63*, AMA 65*, MACN-Ma 31194*, AMA 67*, AMA69*, AMA 74*, AMA 75*, AMA 77*, AMA 81*, AMA 89*, MACN-Ma 31191*, MACN-Ma 31188*, AMA 112, MACN-Ma 31193, MACN-Ma 31200*, AMA 129*, AMA 143*, MACN-Ma 31205 *, MACN-Ma 31189*, AMA 151*, MACN-Ma 31198*, MACN-Ma 31196*, AMA 117, MACN-Ma 31202, AMA 121*, AMA 130*, MACN-Ma 31204*, MACN-Ma 31199*, AMA 150*, AMA 155*, MACN-Ma 31195.

*Molosus fluminensis* (*n* = 12) ♂: MACN-Ma 31144, MACN-Ma 31147, MACN-Ma 31148*, MACN-Ma 31149*, MACN-Ma 31150*, MACN-Ma 31151, MACN-Ma 31152*, MACN-Ma 31153*. ♀: MACN-Ma 31145*, MACN-Ma 31146*, MACN-Ma 31154, MACN-Ma 31155.
