# Supplementary material for: Wing Morphology, Foraging Strategies, and Flight Performance in Six Sympatric Species of Molossid Bats (Chiroptera: Molossidae) from Argentina
Source: Integr Org Biol. 2025 Nov 14;7(1):obaf044. doi: 10.1093/iob/obaf044 (PMC12690267; doi:10.1093/iob/obaf044)
Supplement: obaf044_Supplemental_Files [file obaf044_supplemental_files.zip › Supplementary Material 1-Argoitia et al. 2025.docx]

**Supplementary Material 1**

**Table 1.** Results of Phylogenetic Generalized Least Squares (PGLS) for the six species of molossid bats studied. Asterisk, significant after 10000 rounds of permutation test.

| **Model shape ~ log 10 (CS)** | | | | | | | |
| --- | --- | --- | --- | --- | --- | --- | --- |
|  | *Df* | *SS* | *MS* | *Rsq* | *F* | *Z* | Pr(>*F*) |
| logCS | 1 | 0.00041394 | 0.00041394 | 0.45503 | 33.398 | 1.643 | **0.0497*** |
| Residuals | 4 | 0.00049576 | 0.00012394 | 0.54497 |  |  |  |
| Total | 5 | 0.00090970 |  |  |  |  |  |
| **Model shape ~ log 10 (CS) + flight group (guilds)** | | | | | | | |
|  | *Df* | *SS* | *MS* | *Rsq* | *F* | *Z* | Pr(>*F*) |
| logCS | 1 | 0.00041394 | 0.00041394 | 0.45503 | 45.376 | 18.713 | **0.02475*** |
| Guilds | 1 | 0.00022209 | 0.00022209 | 0.24413 | 24.345 | 14.060 | 0.07895 |
| Residuals | 3 | 0.00027367 | 0.00009122 | 0.30084 |  |  |  |
| Total | 5 | 0.00090970 |  |  |  |  |  |

References: *DF*, degree of freedom; *SS*, squared sum; *MS*, mean squared sum; *Rsq*, coefficient of determination; *F*, *F* – statistic; *Z*, *Z* – statistic; *Pr(>F)*, *p* – value for.


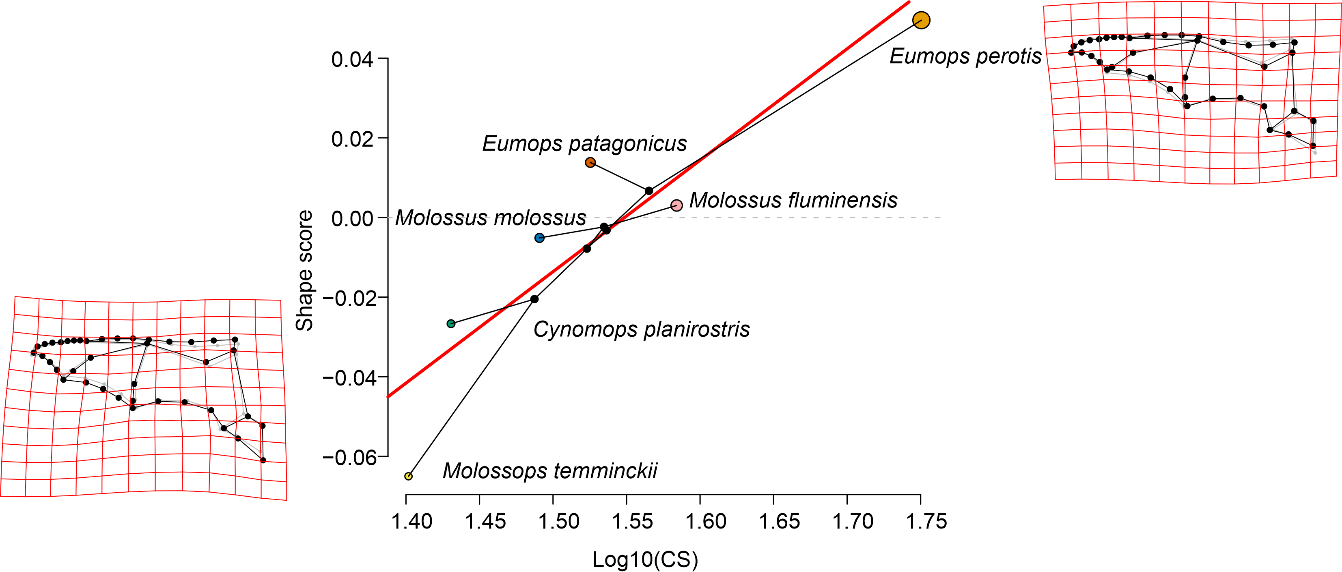


**Figure 1.** Phylogenetic Generalized Least Squares (PGLS) regression of shape against log10-trasnformed centroid size for the six species of molossid bats. Deformation of thin plate spline gridlines plus landmarks and wireframe (gray, consensus) of negative and positive most shape score values are exaggerated three times.
