# Supplementary material for: Wing Morphology, Foraging Strategies, and Flight Performance in Six Sympatric Species of Molossid Bats (Chiroptera: Molossidae) from Argentina
Source: Integr Org Biol. 2025 Nov 14;7(1):obaf044. doi: 10.1093/iob/obaf044 (PMC12690267; doi:10.1093/iob/obaf044)
Supplement: obaf044_Supplemental_Files [file obaf044_supplemental_files.zip › Supplementary Material 2-Argoitia et al. 2025.docx]

**Supplementary Material 2**

Comparison of Regression Analysis performed on both the six and four species data sets.

The multivariate OLS regressions of four species data set between shape and log10-transformed CS was significant, and explaining 5.01% of the variation in shape (allometric scaling) with total sum of squares (SS) = 0.2923 and a residual SS =0.2777. In both morphospaces the species were distributed along a gradient with the smallest on the negative values to the largest on the positive values of the y-axis (shape score).

**
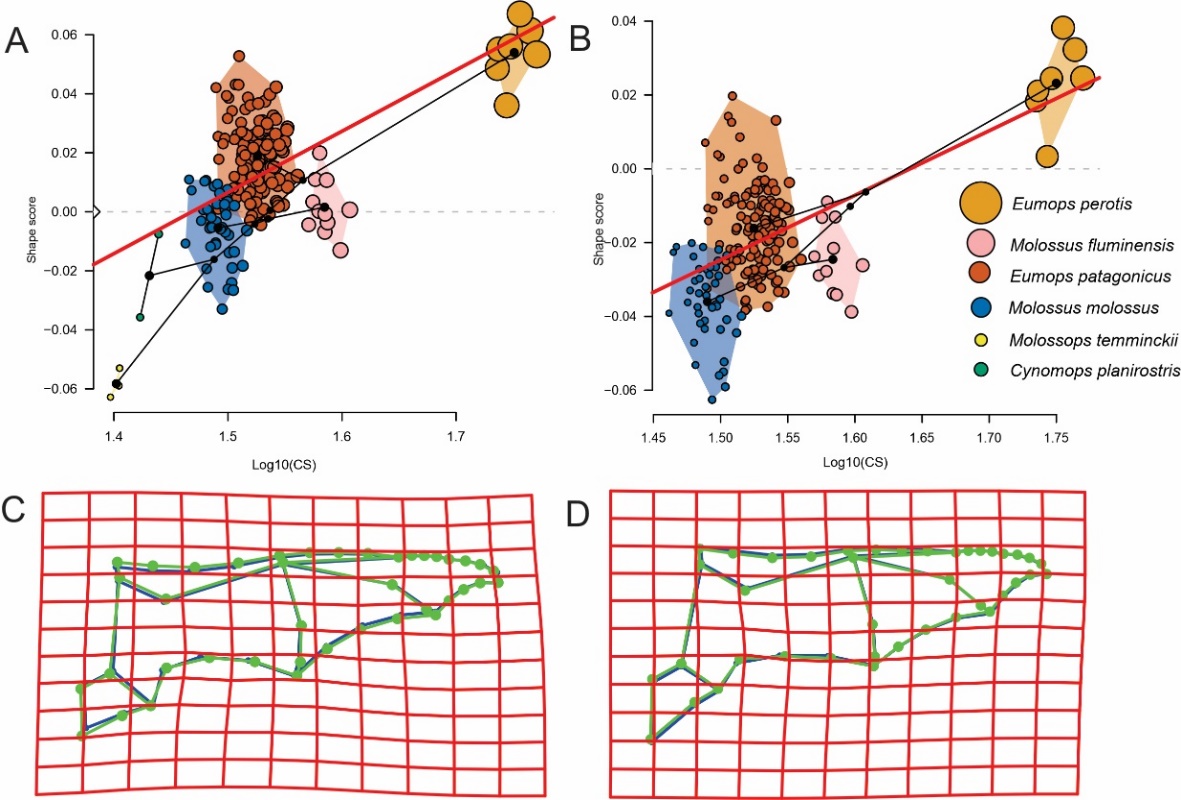
**

**Figure 1.** Multivariate ordinary least squares (OLS) regression analysis of Procrustes coordinates (shape scores) vs. log10-transformed centroid size (CS). The morphospace and projected phylomorphospace of regression analysis including the six species of molossid bats (A; i.e., main text); and the regression analysis including only the four species with more than five specimens (B). The thin plate spline gridlines plus landmarks and wireframe comparing both analysis (six spp. in blue and four spp. in green) of negative (C) and positive (D) shape scores are exaggerated three times.

**Table 1.** Descriptive statistics of Procrustes residuals for the four shared species between both regression morphospaces (six vs four spp. data sets)

| **Species** | **Mean** | **Min** | **Max** |
| --- | --- | --- | --- |
| *Eumops patagonicus* | 0.00464 | 0.00023 | 0.0169 |
| *Eumops perotis* | 0.00269 | 0.00086 | 0.00381 |
| *Molossus molossus* | 0.00508 | 0.00015 | 0.01748 |
| *Molossus fluminensis* | 0.00919 | 0.00088 | 0.01859 |
