# Supplementary material for: Wing Morphology, Foraging Strategies, and Flight Performance in Six Sympatric Species of Molossid Bats (Chiroptera: Molossidae) from Argentina
Source: Integr Org Biol. 2025 Nov 14;7(1):obaf044. doi: 10.1093/iob/obaf044 (PMC12690267; doi:10.1093/iob/obaf044)
Supplement: obaf044_Supplemental_Files [file obaf044_supplemental_files.zip › Supplementary Material 3-Argoitia et al. 2025.docx]

**Supplementary Material 3**

Comparison of Principal Component Analysis performed on both the six and four species data sets.

The PCA of four species data set resulted in the first seven PCs accounting for the 80.34% of cumulative variance. Accordingly, to the *getMeaningfulPCs* function report (see Fig. 1), only the four first axes were find to be meaningful after Bookstein´s (2014) method and used to compare the wing variation with the analysis of the six species data set. As it can be seen by the sedimentation plots, both analyses showed a close similar pattern of accounted variance for each PC (Figure 1).


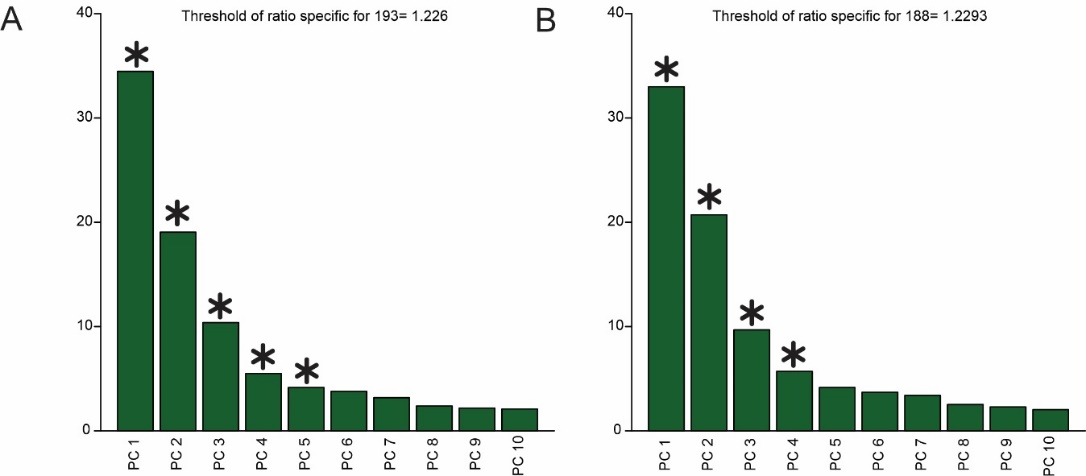


**Figure 1.** Sedimentation plots of PCA results plus Bookstein’s analyses; A, for six species data set (i.e., main text); B, for four species data set (i.e., only those with more than five individual sampled). References: asterisk indicted PC’s interpretable after Bookstein´s (2014) method.

The components PC1 and 2, explained ca. 54% of total variance in both PCA (Fig. 2) and each axis showed the same shape changes in the wings. Both analyses also shared the structure of the morphospace, as well as, the extreme shapes of each axis depicted as TPS grids showing changes relative to the consensus. Same can be seen in the morphospace depicted by PC3 and 4 (Fig. 3). The correlation in a symmetric Procrustes rotation of these four axis (i.e., PC1 to 4) of the shared specimens between the two analyses was very high and significant after 10,000 rounds of permutation test (*r*= 0.9982; *p*-value <0.001). The mean Procrustes residuals between the four first component of these morphospaces (i.e., ca. 70% of cumulative variation) showed to be very low (Table 1). In summary, all these results indicate a very high congruence between these multivariate spaces.

**Table 1.** Descriptive statistics of Procrustes residuals for the four shared species between both PCA morphospaces (six vs four spp. data sets)

| **Species** | **Mean** | **Min** | **Max** |
| --- | --- | --- | --- |
| *Eumops patagonicus* | 0.00328 | 0.00031 | 0.01352 |
| *Eumops perotis* | 0.00480 | 0.00319 | 0.00687 |
| *Molossus molossus* | 0.00447 | 0.00137 | 0.01077 |
| *Molossus fluminensis* | 0.00405 | 0.00130 | 0.01158 |


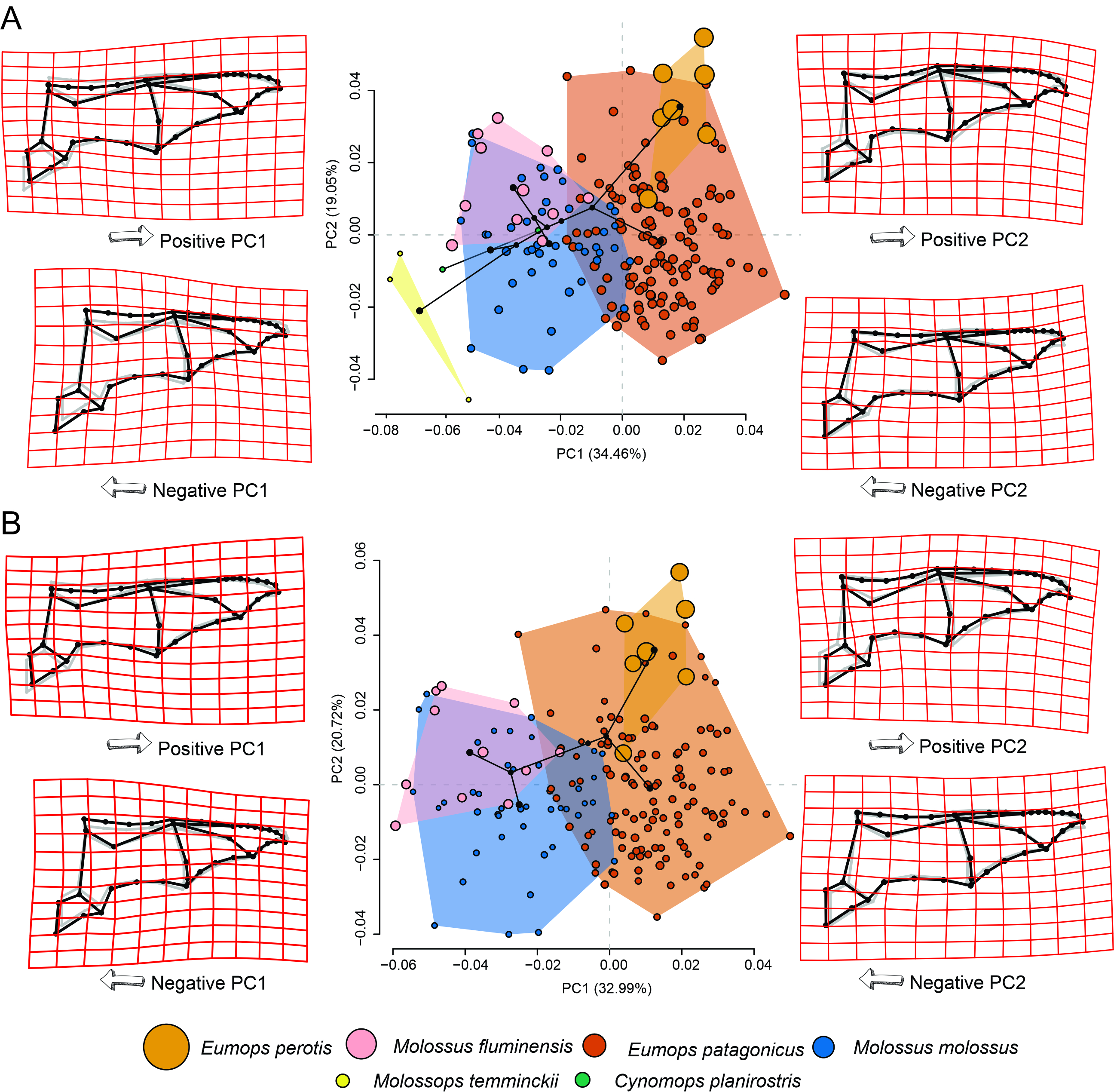


**Figure 2.** Principal Component Analysis (PCA) of six species (A) and four species (B) data sets of molossid bats. Morphospace (center) depicted by the first two principal components (PC1 and PC2). References: the size of the points refers are proportional to the centroid size (CS). Thin plate spline gridlines plus landmarks and wireframe (gray, consensus; i.e., zero values) of negative (below) and positive (above) most first pair of PCs (PC1 (left) and PC2 (right).


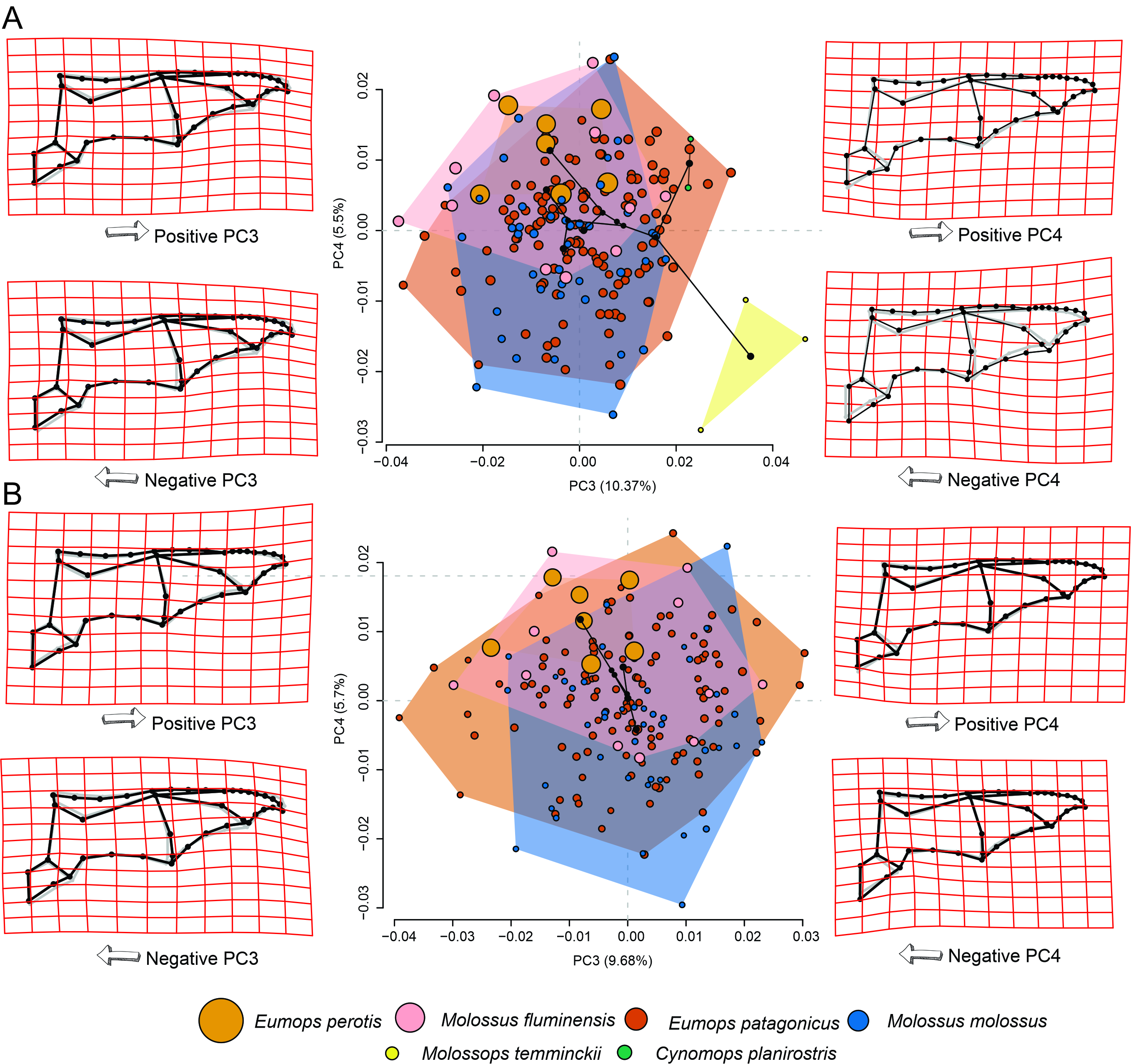


**Figure 3.** Principal Component Analysis (PCA) of six species (A) and four species (B) data sets of molossid bats. Morphospace (center) depicted by the third and four principal components (PC3 and PC4). References: the size of the points refers are proportional to the centroid size (CS). Thin plate spline gridlines plus landmarks and wireframe (gray, consensus; i.e., zero values) of negative (below) and positive (above) most first pair of PCs (PC3 (left) and PC4 (right).
